# Retinal Phenotype in the rd9 Mutant Mouse, a Model of X-Linked RP

**DOI:** 10.3389/fnins.2019.00991

**Published:** 2019-09-19

**Authors:** Antonio Falasconi, Martina Biagioni, Elena Novelli, Ilaria Piano, Claudia Gargini, Enrica Strettoi

**Affiliations:** ^1^Institute of Neuroscience, National Research Council (CNR), Pisa, Italy; ^2^Sant’Anna School of Advanced Studies, Pisa, Italy; ^3^Department of Pharmacy, University of Pisa, Pisa, Italy

**Keywords:** Retinitis Pigmentosa, sprouting, remodeling, retinal pigment epithelium, cone photoreceptor(s), bipolar cell, horizontal cell

## Abstract

Retinal degeneration 9 (rd9) mice carry a mutation in the retina specific “Retinitis Pigmentosa GTPase Regulator (RPGR)” Open Reading Frame (ORF) 15 gene, located on the X chromosome and represent a rare model of X-linked Retinitis Pigmentosa (XLRP), a common and severe form of retinal degeneration (Wright et al., 2010; Tsang and Sharma, 2018). The rd9 RPGR-ORF15 mutation in mice causes lack of the protein in photoreceptors and a slow degeneration of these cells with consequent decrease in Outer Nuclear Layer (ONL) thickness and amplitude of ERG responses, as previously described (Thompson et al., 2012). However, relative rates of rod and cone photoreceptor loss, as well as secondary alterations occurring in neuronal and non-neuronal retinal cell types of rd9 mutants remain to be assessed. Aim of this study is to extend phenotype analysis of the rd9 mouse retina focusing on changes occurring in cells directly interacting with photoreceptors. To this purpose, first we estimated rod and cone survival and its degree of intraretinal variation over time; then, we studied the morphology of horizontal and bipolar cells and of the retinal pigment epithelium (RPE), extending our observations to glial cell reactivity. We found that in rd9 retinas rod (but not cone) death is the main cause of decrease in ONL thickness and that degeneration shows a high degree of intraretinal variation. Rod loss drives remodeling in the outer retina, with sprouting of second-order neurons of the rod-pathway and relative sparing of cone pathway elements. Remarkably, despite cone survival, functional defects can be clearly detected in ERG recordings in both scotopic and photopic conditions. Moderate levels of Muller cells and microglial reactivity are sided by striking attenuation of staining for RPE tight junctions, suggesting altered integrity of the outer Blood Retina Barrier (BRB). Because of many features resembling slowly progressing photoreceptor degeneration paradigms or early stages of more aggressive forms of RP, the rd9 mouse model can be considered a rare and useful tool to investigate retinal changes associated to a process of photoreceptor death sustained throughout life and to reveal disease biomarkers (e.g., BRB alterations) of human XLRP.

## Introduction

Retinitis pigmentosa (RP) is a family of clinically analogous disorders, featuring photoreceptor degeneration and alterations in retinal pigment epithelium (RPE), in most cases leading to blindness (Hartong et al., 2006). Etiology is linked to a plethora of mutations affecting more than 70 genes, involving virtually all aspects of photoreceptor structure and function (Daiger et al., 2007; Wright et al., 2010). These extremely specialized cells have a high metabolic demand, require a complex gene network and rely on a fully functional RPE to sustain the energetic and physiological burden of phototransduction, outer segment renewal and continuous communication with inner retinal neurons (Wright et al., 2010). While the outer segment of photoreceptors is tightly packed with membranes and proteins necessary to phototransduction, the inner segment acts as biosynthetic factory to produce needed proteins and fatty acids. The continuous flow of these fundamental factors to the outer segments relies on a highly specialized connecting cilium (Besharse et al., 1977; Wolfrum and Schmitt, 2000; Burgoyne et al., 2015; Chadha et al., 2019) and it is not surprising that almost one quarter of known photoreceptor degeneration-causing genes are involved in the function of this organelle (RetNet, the Retinal Information Network)^1^. The Retinitis Pigmentosa GTPase Regulator (RPGR) is located on the X chromosome and, together with its interactome (Zhang et al., 2019), plays a critical role for connecting cilium function (Megaw et al., 2015). Mutations in RPGR account for 10–20% of all RP cases and 70–80% of all cases of X-linked RP (Huang et al., 2012; Megaw et al., 2015; Tsang and Sharma, 2018) with different mutations corresponding to different retinal phenotypes (Megaw et al., 2015; Charng et al., 2016; Lyraki et al., 2016). RPGR exists in two different isoforms: one is expressed throughout the body, while the other is retina-specific. The retinal-specific form is composed of 15 exons, the first 14 of which shared with the non-retinal isoform. Exon 15 or Open Reading Frame 15 (ORF15) is solely present in retinal RPGR and constitutes a mutational hotspot in the gene (Megaw et al., 2015; Lyraki et al., 2016; Rao et al., 2016).

The rd9 mouse model carries a 32 bp duplication in ORF15 with a premature stop-codon, causing absence of the protein and a slowly progressing loss of photoreceptors (Thompson et al., 2012). The main features of this rare model of X-linked RP have been described in previous studies. Nonetheless, the relative rate of degeneration of rods and cones, as well as possible remodeling of inner retinal neurons and RPE following the slow pattern of photoreceptor death typical of this model, remain unknown. Yet, evaluation of retinal effects beyond photoreceptors are of utmost importance, especially in view of newly developed potential therapeutic strategies, such as gene-therapy or epiretinal prostheses, which rely considerably upon preservation of retinal architecture.

In this study, we provide a secondary retinal characterization of the rd9 mouse model, focusing on cells directly interacting with photoreceptors, and namely bipolar and horizontal cells, as well as on non-neuronal retinal cell types (Muller cells, microglia/macrophages and the, RPE), describing their morphological changes in parallel to photoreceptor loss and to functional abnormalities detected by ERG recordings.

## Materials and Methods

### Mouse Lines and Animals Used

Animals were treated in accordance to Italian and European institutional guidelines, following experimental protocols approved by the Italian Ministry of Health (Protocol #17/E-2017, Authorization 599 2017-PR, CNR Neuroscience Institute, Pisa; Protocol #DGSAF0001996/2014, Authorization 653/2017-PR, Department of Pharmacy, University of Pisa) and by the Ethical Committees of both Institutions. Protocols adhere to the Association for Research in Vision and Ophthalmology (ARVO) statement for the use of animals in research.

Male rd9/Y, female rd9/X and WT mice were used for this study. Rd9 mice are naturally occurring mutants, identified by the Jackson Laboratories (Chang et al., 2002), and have a C57Bl6/J background. All mice were originally from Jackson (Bar Harbor, Maine, United States). Groups of *n* = 4 mice were used for studies carried on at specific ages (12 months for WT mice, 12 and 18 months for rd9 mice) for both quantitative and qualitative analysis. Additional rd9/Y male mice (*n* = 4) were used for pilot experiments. A total of 16 rd9 and 8 WT mice were used for morphological studies only. A group of 12-months old male rd9/Y and female rd9/X mice (*n* = 9) and 12 months old WT (*n* = 5) were used for ERG recordings and their retinas further studied by morphological methods.

### Tissue Preparation, Histology and Immunocytochemistry (ICCH)

Mice were anesthetized with intraperitoneal injections of 3-bromo-ethanol in 1% tert-amyl alcohol (Avertin, 0.1 ml/5 g body weight), their eyes enucleated. Animals were killed by cervical dislocation or intracardiac Avertin injection. Eyes were dorsally labeled and eye cups were obtained by removing anterior segments and lens and fixed in 4% paraformaldehyde (PFA) in 0.1 M phosphate buffer, PB, pH7.4, for 1 h, at room temperature. Afterward, they were washed four times (15’ intervals) in PB and infiltrated in 30% sucrose in PB overnight. Eye cups were then frozen in Tissue-Tek O.C.T. compound (4583, Sakura Olympus, Italy) using cold isopentane (−80°C) and keeping a reference on the dorsal pole. Cryostat sections (12 μm thick) were collected on Super Frost slides and used for immunocytochemistry (ICCH). Some eyes were used to prepare retinal whole mounts, in which the retina was separated from the RPE and flattened by making four radial cuts toward the head of the optic nerve. For some eyes, the RPE was also used for ICCH as described below. ICCH on retinal sections, whole mounts and RPE was performed following (Barone et al., 2012), by incubation in (i) blocking solution with 0.3% Triton-X 100, 5% of the serum of the species in which the secondary antibody was generated and 0.01 M Phosphate Buffer Saline (PBS); incubation time was 2 h for the sections and overnight for whole mounts and RPEs; (ii) primary antibody (Ab), diluted in PBS, 0.1% Triton-X 100 and 1% serum; incubation time was overnight for the sections and 3 days for whole mounts and RPEs; (iii) fluorescent secondary Ab, diluted as the primary Ab; incubation time was 2–3 h for the sections and 2 days for whole mounts and RPEs. Incubations steps were done at 4°C. Mouse monoclonal, primary Abs used for retinal sections were against: Neurofilament 200 (13552 AbCam, Cambridge United Kingdom; diluted 1:400), Ctbp2/Ribeye (BD Transduction Laboratories, Milan, Italy; diluted 1:500), Post-Synaptic Density 95 (13552 AbCam, Cambridge United Kingdom; diluted 1:500); Light Sensitive Channel (kindly donated by Robert Molday, University of British Columbia, Vancouver, Canada), diluted 1:1000); Protein Kinase Cα (PKCα; P5704, Sigma-Aldrich, Italy; diluted 1:800), Synaptotagmin 2 (ZNP-1) (Zebrafish International Resource Center, Eugene, OR, United States; diluted 1:500). Rabbit polyclonal, primary Abs were: Cone Arrestin (AB15282, Merck-Millipore, Italy; diluted 1:5000); Calbindin D (CB38a, Swant Ltd., Switzerland, diluted 1:500); Protein Kinase Cα (PKCα; P4334, Sigma-Aldrich, Italy; diluted 1:800); Glial Fibrillary Acidic Protein (GFAP; G9269, Sigma-Aldrich, Italy; diluted 1:1000), Iba1 (019-19741, Wako, United States; diluted 1:500), Zonula Occludens 1 (ZO-1) (ZYMED Laboratories 40_2300, diluted 1:100). Sheep (polyclonal or monoclonal) primary Abs were: Secretagogin (SCGN) (BioVendor, GmbH, Germany, diluted 1:2000). Abs against Cone Arrestin were also used in whole mount preparations, at 10x the concentrations used for sections. Secondary antibodies were: donkey anti-mouse Alexa Fluor 488 (A-21202, Life Technologies, Italy); donkey anti-rabbit Rhodamine Red X (715296151); Donkey Anti-Sheep (713-546-147), all from Jackson ImmunoResearch laboratories, United States; these were diluted 1:1000 for retinal sections and for whole mount preparations. For nuclear counterstaining, retinal sections were incubated for 2 min in Hoechst (33342), from Life technologies, Italy, diluted 1:1000. After rinsing in PBS, specimens were mounted in Vectashield (H-1000; Vector Laboratories, Burlingame, CA, United States) and coverslipped.

#### RPE Preparation

After separation of the retina from the outer eye (RPE and sclera), the sclera was carefully made clear of all muscle insertions. The outer ocular layers were then radially cut with 4 to 12 incisions toward the head of the optic nerve and incubated (RPE side up) in small plastic wells, processed for ICCH as described above and mounted flat on glass slides.

#### Imaging

Images of retinal preparations were obtained with a Zeiss Imager.Z2 microscope equipped with an Apotome2 device (Zeiss, Milan, Italy), using a Plan Neofluar 40x/1.25 and a Plan Neofluar 63x/1.25 oil objectives. Images were saved as tiff files; brightness and contrast were adjusted with the Zeiss software ZEN^®^PRO 2012 or with Adobe Photoshop. Retinal whole mounts were also imaged with the Imager.Z2 microscope using EC Plan-Neofluar 5x/0.16 M27, 10x/0.3 M27 and 20x/0.50 M27 objectives; images were tiled with ZEN module “Tiles & Positions” software to reconstruct the entire retinal surface when needed.

### Outer Nuclear Layer (ONL) Measurements on Retinal Sections

Three equatorial retinal sections (including the optic nerve head) were obtained from eyes of different mice at each age point; after nuclear staining, 4 *z*-stack images (6 μm thickness, 0.6 μm intervals) at different eccentricities (peripheral ventral, central ventral, peripheral dorsal, central dorsal) were acquired from each section. Using the count tool of Adobe Photoshop, the number of ONL rows were counted in each image and the results were averaged to obtain the mean number (and standard error) of ONL rows per group (WT 12 months, rd9 12 months and rd9 18 months). The coefficient of variation (standard deviation/mean) was calculated for each eccentricity in each retina and results were averaged to obtain the mean Coefficient of Variation per experimental group (WT 12 months, rd9 12 months, and rd9 18 months).

### Cone Counts in Retinal Whole Mounts

Whole-mount retinas were stained for cone Arrestin. To assess total cone numbers taking into account local anisotropies in retinal degeneration patterns and center-to-periphery changes in cell density, cells were counted in 16 fields regularly spaced along the two main (horizontal and vertical) retinal meridians (8 fields along the dorso-ventral axis and 8 along the naso-temporal axis, respectively), covering the retina from the far periphery to the proximity of the optic nerve head. Counting fields were 223.8 × 167.6 μm^2^ fields within which z-stacks of 3 focal planes at 0.6 μm intervals, (1.8 μm total thickness) encompassing cone inner segments were obtained. Maximum projections of *z*-stacks were used for further analysis.

#### Manual Counting

The average number of cones per image was assessed as described before (Barone et al., 2012). The number of cones/mm^2^ in each retina was then estimated. The Adobe Photoshop Measure tool was used to measure the area of each retina using low-magnification images of whole mounts. Then, the number of cones per retina were estimated and averaged to obtain the mean number of cones per retina in each experimental group. Coefficient of variation (standard deviation/mean) was calculated for each eccentricity level in each retina for all experimental groups.

#### Automated Counting

Images used for manual counting were also used to test a method for automated counting of cones developed *ad hoc*. Through a custom-made MATLAB^®^ (Mathworks, United States) script, images were binarized; connected components in the image were identified and those with an area smaller than 2% of the average cone’s area were not considered for further analysis. The image was then down sampled with a factor 0.3 and watershed segmentation was applied to ensure appropriate counting of touching components. As in the manual counting, all components intersecting the right or bottom borders were not included in the counts. After obtaining the number of cones in each acquired image, the number of cones per retina, the mean number of cones per retina in each experimental group as well as the individual and mean value of the coefficient of variation were calculated as for the manual counting protocol.

Parameters for area filtering (2%) and down-sampling (0.3) were identified empirically based on accuracy of the algorithm on test images. Performance assessment of the algorithm was carried out by Pearson correlation analysis between manual and automated counting in single images and through analysis of the error (*Manual  result  −  Automated  Result)/  Manual  result*) distribution. Results are shown in Supplementary Figure S1. The automated method was found to have an accuracy of 95% and allowed much faster counting of elements in each image.

### ZO-1 Staining Intensity Quantification in RPE

Each whole-mount RPE was stained for ZO-1 and revealed with a secondary antibody conjugated with Alexa Fluor 488. *z*-stack images (2.5 μm thickness) were acquired with the Zeiss Imager.Z2 microscope equipped with an Apotome2 device, using both the green and red filters, which made numerous autofluorescent bodies visible in the *cytoplasm* of RPE cells (Supplementary Figure S2), as previously reported (Marmorstein et al., 2002). Acquisition parameters were set for the first rd9 12 months old sample and then kept consistent for images from all different preparations.

To understand whether *cytosolic* fluorescence (Supplementary Figure S2) had to be attributed to non-specific secondary antibody binding or to effective presence of ZO-1 (revealed by Alexa Fluor 488-s) we performed spectral profiling of RPE cells using a lambda scan routine of a Leica TCS SL confocal microscope (Leica Microsystems, Milan, Italy) equipped with an argon and a helium/neon laser, using a 40x/1.25 HCX PL APO oil objective. Emission spectra with an excitation wavelength of 488 nm allowed univocal differentiation of specific and non-specific staining (Supplementary Figure S2), confirming that cytosolic labeling, observed with both green and red filters through the standard fluorescence microscope, corresponded to autofluorescent material, well different from Alexa Fluor 488-conjugated secondary Ab in terms of emission spectra. Based on these findings, fluorescence intensity in the green channel (containing ZO-1 positive elements) was quantified with the aid of a custom-made MATLAB script, discarding pixels with a fluorescence intensity higher than 8 arbitrary units in the red channel, allowing customized denoising of the pictures. Nonetheless, consistent parameters used during acquisition caused saturation of the ZO-1 signal in the WT, biasing downward fluorescence intensity measurement of WT ZO-1. This fact makes our quantification indicative and not absolute.

### ERG Recordings

ERGs were recorded from dark-adapted mice by standard methods (Gargini et al., 2007). Briefly, coiled gold electrodes were placed in contact with the cornea moisturized by a thin layer of gel. Pupils were fully dilated by application of a drop of 1% atropine (Farmigea, Pisa, Italy). Scotopic ERG recordings were average responses (*n* = 5) to flashes of increasing intensity (1.7 × 10^–5^ to 377.2 cd^∗^s/m^2^, 0.6 log units steps) presented with an inter-stimulus interval ranging from 20 s for dim flashes to 1 min for the brightest flashes. Cone (photopic) components were isolated by superimposing the test flashes (0.016 to 377.2 cd^∗^s/m^2^, 0.6 log units steps) on a steady background of saturating intensity for rods (30 cd/m^2^), after at least 15 min from background onset. Amplitude of the a-wave was measured at 7 ms after the onset of light stimulus; amplitude of the b-wave was measured from the peak of the a-wave to the peak of the b-wave. Oscillatory potentials (OPs) were also measured in both scotopic and photopic conditions. OPs were extracted digitally by using a fifth-order Butterworth filter as previously described (Hancock and Kraft, 2004; Lei et al., 2006). Peak amplitude of each OP (OP1–OP4) was measured (Piano et al., 2016). ERG data were collected from 5 WT and 8 rd9 mice, respectively.

### Statistics and Data Analysis

Statistical comparisons were run on GraphPad Prism v6 (GraphPad Software, San Diego, CA, United States) after ensuring the data passed a normality distribution test. Comparisons were made using a double-tailed *t*-test analysis, with a confidence interval (CI) of 95%. Statistical significance was assessed through *p*-values reported as asterisks in graphs (^∗∗∗^ for *p* ≤ 0.001, ^∗∗^ for *p* ≤ 0.01, ^∗^ for *p* ≤ 0.05). All results are shown as mean ± standard error of the mean, unless otherwise specified.

## Results

### Photoreceptor Degeneration

**FIGURE 1:**
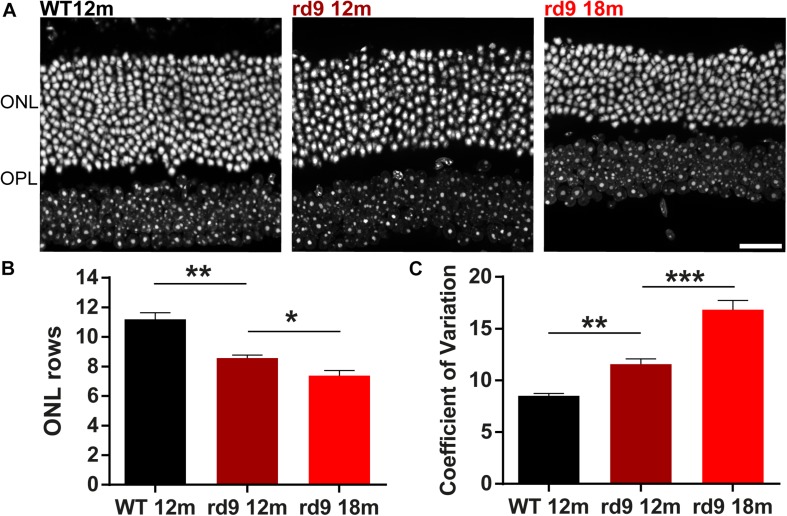
Outer nuclear layer (ONL) in rd9 mutants. **(A)** Representative images of vertical retinal sections with nuclear staining in WT 12 months old, rd9 12 months old and rd9 18 months old mice. Scale bar is 20 μm. **(B)** Quantification [mean ± standard error (se)] of ONL rows in WT 12 months old, rd9 12 months old and rd9 18 months old mice. **(C)** Quantification (mean ± se) of Coefficient of variation in ONL rows (Coefficient of Variation) in WT 12 months old, rd9 12 months old and rd9 18 months old mice. ^∗∗∗^for *p* < 0.0001, ^∗∗^for *p* < 0.005, ^∗^for *p* < 0.05.

Global photoreceptor loss in rd9 mice was assessed through counting ONL rows of nuclei in vertical retinal sections (Figure 1) and counting cones in whole-mount retinas stained for cone arrestin, to confirm and extend previous analysis of ONL thickness in this mutant (Thompson et al., 2012).

ONL rows are slightly but clearly diminished in rd9 12 months old mice (from now on referred to as “rd9 12 months”) (8.6 ± 0.2 rows) compared to age matched WT (11.2 ± 0.3 rows) (*p* = 0.003); photoreceptor loss continues in rd9 18 months old mice (from now on referred to as “rd9 18 months”) (7.4 ± 0.2 rows) with respect to rd9 12 months (*p* = 0.03) (Figures 1A,B), confirming previous findings (Thompson et al., 2012). We could not detect a visible topographical pattern in the loss of photoreceptors, which, however, upon quantitative analysis, exhibits a “patchy” distribution with some, apparently random, areas displaying fewer photoreceptors than others. We measured the extent of irregularity in the process of rod degeneration by measuring the coefficient of variation (standard deviation/mean) of ONL rows (Figure 1C). Indeed, rd9 12 months showed a significantly higher coefficient of variation in ONL rows (11.6 ± 0.5) compared to WT controls (8.51 ± 0.2) (*p* = 0.0008), and the pattern become even more irregular in rd9 18 months (16.84 ± 0.9) (*p* < 0.0001). An indication of ON irregularity can be obtained by Figure 9B (arrows).

**FIGURE 2:**
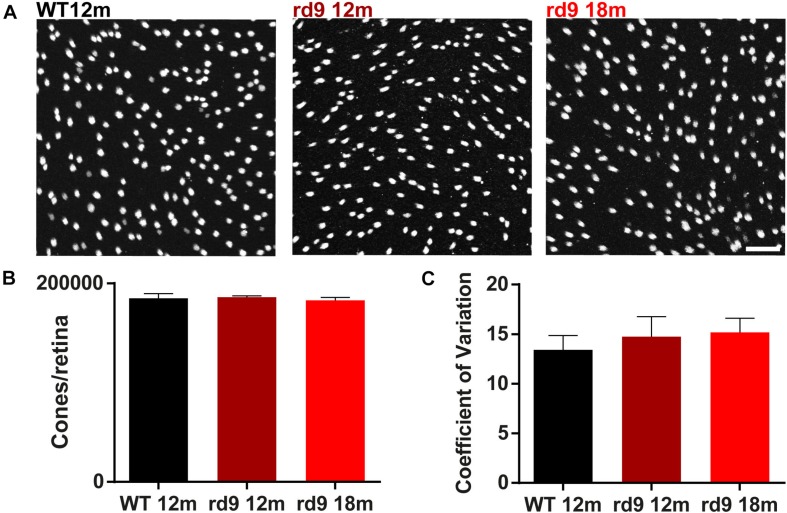
Cone loss in retinal whole mounts. **(A)** Representative images of whole mount ICCH with cone Arrestin antibodies from WT 12 months old, rd9 12 months old and rd9 18 months old mice. Scale bar is 20 μm. **(B)** Quantification (mean ± se) of cones/retina in WT 12 months old, rd9 12 months old and rd9 18 months old mice. **(C)** Quantification (mean ± se) of Coefficient of Variation in cones/retina (Coefficient of Variation) in WT 12 months old, rd9 12 months old and rd9 18 months old mice. Scale bar is 20 μm.

To assess whether the degeneration process affected rods and cone equally, we counted the number of cones/retina using whole mount preparations and also determining the coefficient of variation of these data (Figure 2). Results show no significant loss of cone photoreceptors in both rd9 12 months (186,129.9 ± 1,260.4 cones/retina) and rd9 18 months (182,865.2 ± 2,992.9 cones/retina) compared to WT 12 months (184,921.5 ± 4687.4 cones/retina) (Figures 2A,B). Consistently with the absence of numerical alterations in cone numbers, the coefficient of variation does not vary either between the analyzed experimental groups (WT12 months, 13.4 ± 1.45; rd9 12 months, 14.7 ± 2.0; rd9 18 months 15.2 ± 1.4) (Figure 2C).

**FIGURE 3:**
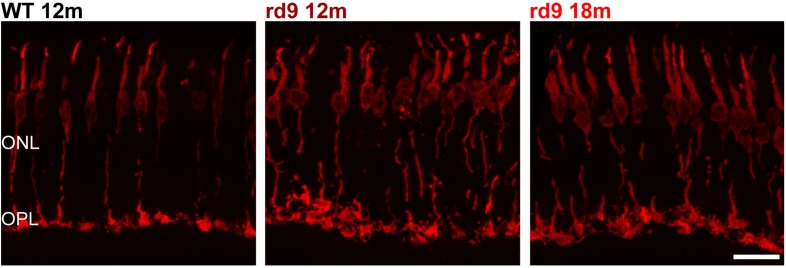
Cone morphology. Representative images of vertical retinal sections stained with anti-cone Arrestin antibodies from WT 12 months old, rd9 12 months old and rd9 18 months old mice. Scale bar is 20 μm. Note the enlargement and slight misalignment of cone pedicles in rd9 compared to WT mice. ONL, Outer Nuclear Layer; OPL, Outer Plexiform Layer.

Besides complete survival of cones, we excluded the occurrence of major morphological alterations in this photoreceptor type (Figure 3), confirming previous data (Thompson et al., 2012). However, an enlargement of cone pedicles and a distribution along a more irregular plane were observed (Figure 3), likely attributable to loss of synaptic spherules and OPL rearrangement following degeneration of rods. Yet, cone pedicles were never observed outside the boundaries of the OPL.

Altogether, our quantitative analysis shows that photoreceptor loss in rd9 mice is mainly due to rod degeneration, while cone number and architecture are mostly preserved, and is associated to intraretinal variation, in agreement with previous findings in humans and murine models of RPGR related disease (Charng et al., 2016). Yet, ERG data (see below) indicate the occurrence of functional abnormalities preceding any morphological alteration in cone cells.

If not otherwise specified, histological images shown to illustrate inner retinal changes were obtained from areas of maximum ONL thinning.

### Remodeling of Horizontal Cells

**FIGURE 4:**
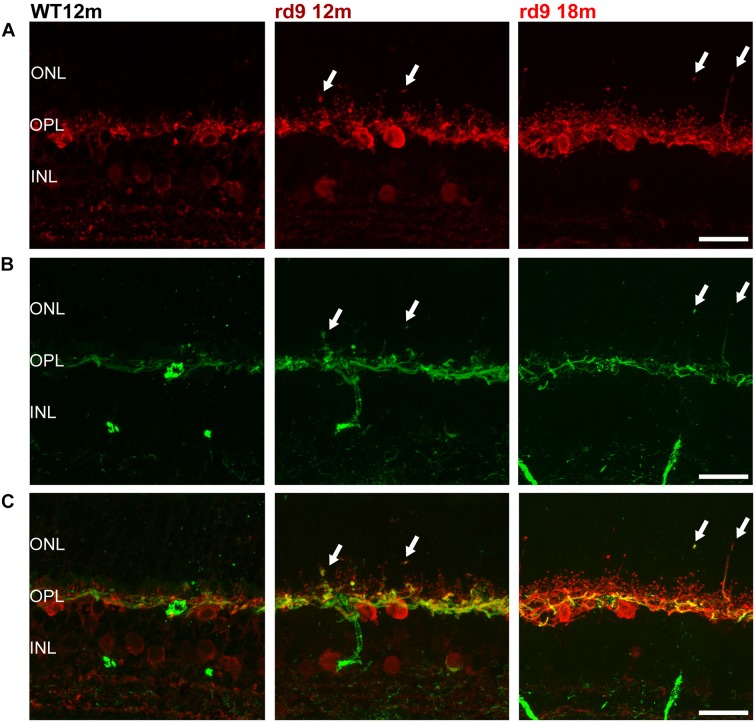
Horizontal cell axonal and dendritic remodeling. **(A)** Representative images of vertical retinal sections stained with anti-Calbindin (red) antibodies from WT 12 months old, rd9 12 months old and rd9 18 months old mice. Scale bar is 20 μm. **(B)** Same fields as above stained with anti-Neurofilament 200 (green) antibodies. Scale bar is 20 μm. **(C)** Merge of **(A)** and **(B)**. Scale bar is 20 μm. Arrows indicate sprouting of the horizontal cell’s rod-connected axonal compartment. ONL, Outer Nuclear Layer; OPL, Outer Plexiform Layer; INL, Inner Nuclear Layer.

Horizontal cells in the mouse retina are a homogenous axon-bearing population. They receive glutamatergic input from photoreceptors, with dendrites being postsynaptic to cones and axonal arbors receiving synaptic inputs from rods. We studied the morphology of these two specific horizontal cell compartments by means of anti-Calbindin D (Figures 4A,C) and anti-Neurofilament 200 antibodies (Figures 4B,C) labeling the entire horizontal cell and its axonal compartment (Gonzälez-soriano, 1994), respectively.

Siding the slow loss of rods, horizontal cells in rd9 retinas sprout profusely with visible remodeling affecting the axonal, rod-connected, components (Figures 4A–C, arrows). Thick sprouts can be followed in the outer retina of rd9 12 months with their thin, apical portions ending deeply in the outer half of the ONL (Figures 4B,C). Progressing from rd9 12 months to rd9 18 months, an increase in length of dendrites of horizontal cells was also observed, with fine processes penetrating the outer retina and becoming less orderly stratified as the degeneration proceeds (Figures 4A,C).

Changes in the axonal compartment of horizontal cells likely reflect the loss of rods, while the subsequent dendritic sprouting suggests abnormalities also in cone-horizontal cell interactions.

### Remodeling of Bipolar Cells

Bipolar cells morphology can be studied with specific antibodies, while co-staining of photoreceptor synaptic contacts provides information of remodeling in the OPL.

**FIGURE 5:**
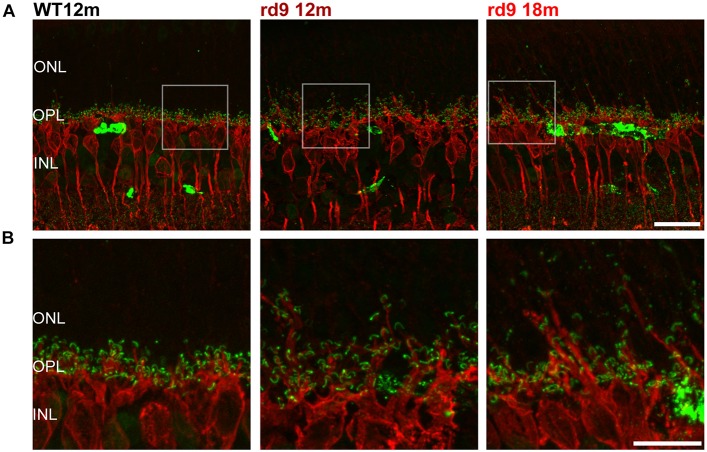
Rod Bipolar cell dendritic remodeling. **(A)** Representative images of vertical retinal sections stained with anti-PKCα (red) and anti-Ribeye (green) antibodies from WT 12 months old, rd9 12 months old and rd9 18 months old mice. Scale bar is 20 μm. **(B)** Details from images in A. Scale bar is 20 μm. Note the extension of bipolar cell dendrites beyond the OPL toward photoreceptor nuclei. ONL, Outer Nuclear Layer; OPL, Outer Plexiform Layer; INL, Inner Nuclear Layer.

To study rod bipolar cells, we used anti-PKCα antibodies while labeling photoreceptor ribbon synapses with anti Ctbp1 (Ribeye) antibodies (Figure 5). Consistently with rod photoreceptor loss, we observed dendritic sprouting of rod bipolar cells in rd9 12 months, with sprouts virtually always abutting ribeye-positive rod synaptic terminals (Figure 5). Fine dendrites of rod bipolar cells elongate toward the ONL, following putative retraction of rod synaptic terminals, and these changes become more evident as the disease progresses (rd9 18 months) and more precisely as rod degeneration continues (Figure 5). The inner aspect of rod bipolar cells (axon and axonal arborizations) appeared identical to the WT counterparts (Figure 5A).

**FIGURE 6:**
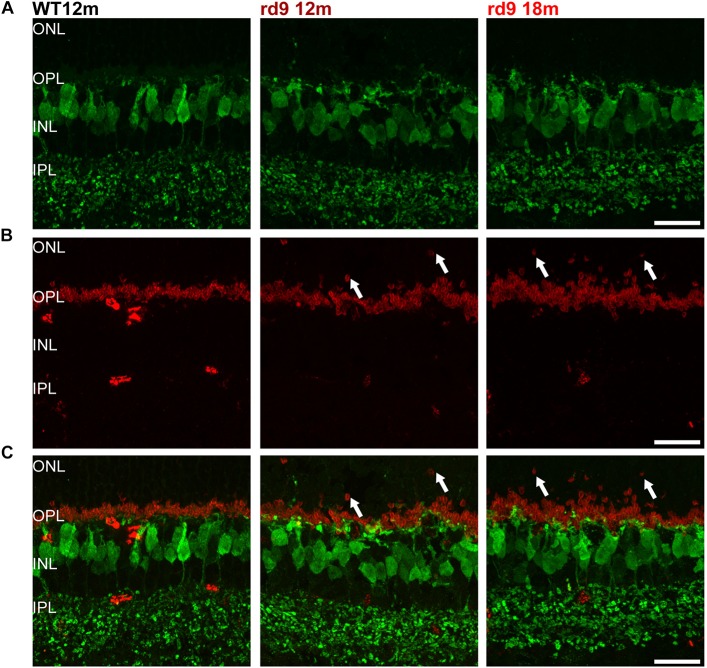
Cone Bipolar cell morphology. **(A)** Representative images of vertical retinal sections stained with anti-Secretagogin (green) antibodies from WT 12 months old, rd9 12 months old and rd9 18 months old mice. Scale bar is 20 μm. **(B)** Same retinal fields as above after staining with Post Synaptic Density 95 (PSD95) (red) antibodies. Scale bar is 20 μm. **(C)** Merge of **(A)** and **(B)**. Scale bar is 20 μm. Note lack of correspondence between displaced rod presynaptic terminals (PSD95 positive) and dendrites of cone bipolar cells (SCGN) (arrows).

**FIGURE 7:**
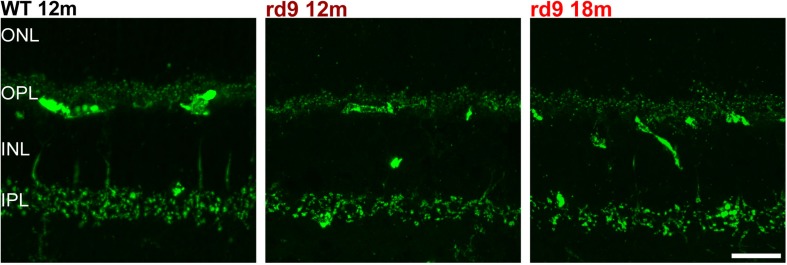
Cone Bipolar cell synaptic contacts. Representative images of vertical retinal sections stained with anti-Synaptotagmin 2 antibodies from WT 12 months old, rd9 12 months old and rd9 18 months old mice. Scale bar is 20 μm. Regular alignment of dendrites in the OPL and axonal arbors in the IPL are evident. ONL, Outer Nuclear Layer; OPL, Outer Plexiform Layer; INL, Inner Nuclear Layer; IPL, Inner Plexiform Layer.

With similar methods, we assessed putative remodeling of cone to cone bipolar cell synapses (Figure 6). Some subtypes of cone bipolar cells (type 2, 3, 4, 5, 6) were stained through anti-secretagogin (SCGN) antibodies (Kim et al., 2008; Puthussery et al., 2010) and their morphology studied, while photoreceptors synaptic contacts were identified by anti-PostSynaptic Density 95 (PSD95) to investigate the presence of paired remodeling, as done for rod bipolar cells (Figures 6A,B). SCGN positive cone bipolar cells did not show dendritic sprouting or abnormality of any kind (Figures 6A,C) and maintained a morphology undistinguishable from that of WT counterparts. Conversely, rod photoreceptor synaptic terminals, labeled by PSD95 antibodies, confirm retraction in the ONL and irregular arrangement in the OPL (Figures 6B,C). Confirming these results, synaptotagmin-2 (ZNP-1) positive cells, which include cone bipolar cell types 2 and 6 (Berntson and Morgans, 2005; Wassle et al., 2009) retained unaltered morphologies in both dendritic and axonal compartments (Figure 7).

Altogether, these observations indicate the occurrence of rod degeneration, moderate remodeling of rod-connected neurons, with sparing of the cone-pathway as the degeneration proceeds.

### Retinal Physiology

**FIGURE 8:**
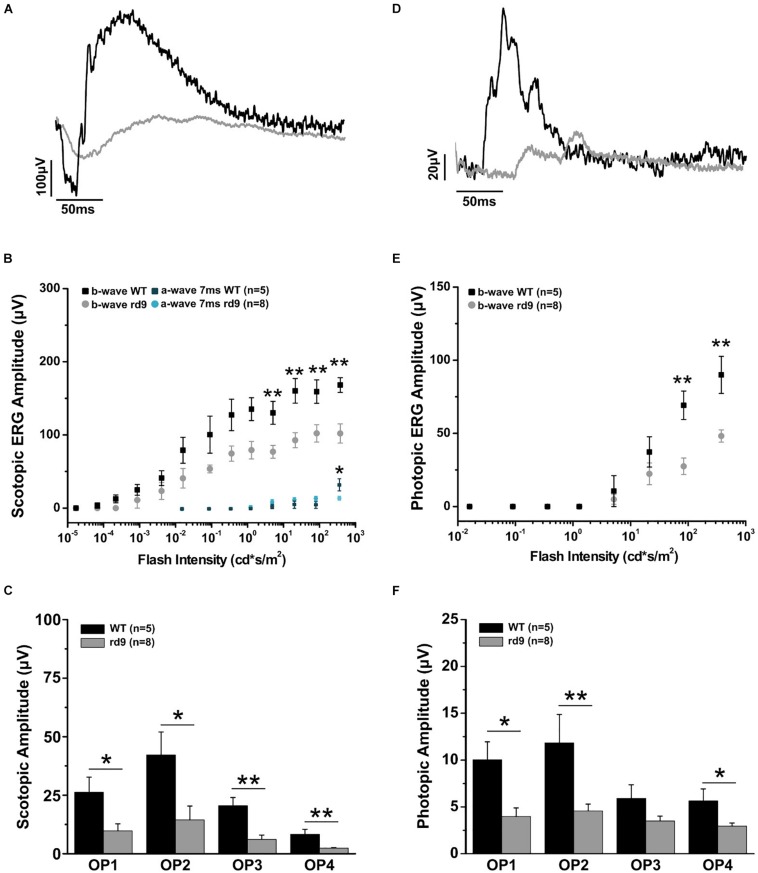
Impairment of scotopic and photopic retinal function. **(A)** Representative scotopic flash ERG recordings from WT and rd9 mice in response to a flash intensity of 377.2 cd^∗^s/m^2^, the highest used for all experiments. **(B)** Scotopic a-wave and b-wave amplitude as a function of flash intensity from WT and rd9 mice. Both a-wave and b-wave amplitudes are significantly reduced in rd9 mutants. **(C)** Scotopic oscillatory potentials amplitude (OP1-OP4) extracted from ERG response to the bright test flash (377 cd^∗^s/m^2^). **(D)** Representative photopic flash ERG recordings from WT and rd9 mice in response to a flash intensity of 377 cd^∗^s/m^2^ superimposed on steady background of 30 cd/m^2^. **(E)** Photopic b-wave amplitude as a function of flash intensity. Cone function is significantly reduced in rd9 mice. The graph shows all the luminances used in the photopic ERG protocol, corresponding to medium and high intensity luminances also used in the scotopic ERG, but superimposed on fixed background of 30cd/m^2^. **(F)** Photopic oscillatory potentials (OP1-OP4) extracted from the ERG response to the bright test flash (377 cd^∗^s/m^2^). OPs show significantly lower amplitudes in rd9 mutants compared to WT mice. ^∗∗^for *p* < 0.005, ^∗^for *p* < 0.05.

Functional analysis of one-year-old rd9 animals showed a reduction of retinal light responses compared to age-matched WT controls mice (Figure 8), confirming previous results in this mouse model (Thompson et al., 2012). The amplitude of the a-wave of the scotopic ERG measured 7 ms after stimulus onset (and directly correlated with the dark current of the photoreceptors) was significantly reduced in rd9 mice; the b-wave amplitude, an indicator of bipolar cell function, also showed a significant decrement. ERG responses following photopic stimulation also showed a significantly reduction in amplitude as well. The kinetic of the response is also slower in rd9 respect to the control mice in both types of ERG protocol used (Table 1). The OPs, extrapolated from both ERG protocols, show a significant reduction in amplitude (Figures 8C,F), demonstrating that not only photoreceptors (OP1) but also neurons of the inner retina (OP2-OP4) are compromised by the degenerative processes.

**TABLE 1:** Peak time values of scotopic and photopic ERG.

**Flash intensity (cd^∗^s/m2)**	**WT 12 months Peak time (mean ± SD)**	**rd9 12 months Peak time (mean ± SD)**	***p*-value**
**Scotopic ERG**			

0.09	0.119650.00617	0.159470.01076	0.013^∗^
0.36	0.096040.00618	0.140270.01637	0.023^∗^
1.29	0.088840.0053	0.126770.0149	0.026^∗^
5.12	0.076070.00565	0.115070.01131	0.013^∗^
21.2	0.071960.00424	0.141870.02364	0.0085^∗∗^
83.7	0.07860.00773	0.11060.01481	N/A
377	0.077340.00384	0.136070.01625	0.004^∗∗^

**Photopic ERG**			

5.12	0.105350.02259	0.152270.01785	N/A
21.2	0.063810.0163	0.095630.01441	N/A
83.7	0.057190.00903	0.080110.01451	N/A
377	0.063590.00697	0.08980.0143	N/A

*^∗∗^for *p* < 0.001, ^∗^for *p* < 0.05.*

### Activation of Muller Glia, Astrocytes and Microglia/Macrophages

Recent data indicate pathological glial cell phenotypes actively contributing to disease progression in the retina (Fletcher et al., 2007; Zhao et al., 2015; Silverman and Wong, 2018; Guadagni et al., 2019) and, more in general, in the CNS (Liddelow et al., 2017; Deczkowska et al., 2018). We examined the morphology of the main glial cell types in the retina of the rd9 mutant, focusing on astrocyte and Muller cell reactivity as assessed by GFAP staining, and on the morphology and distribution of microglia/macrophages.

**FIGURE 9:**
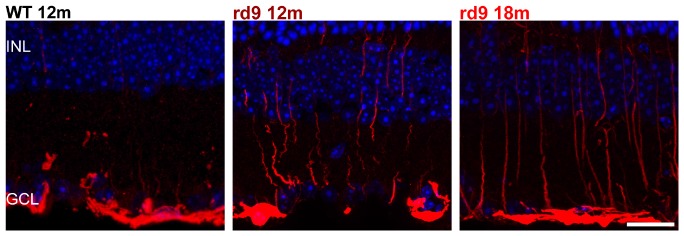
Astrocytes and Muller cells. Representative images of vertical retinal sections labeled with anti-GFAP antibodies (red) and counterstained with DAPI (blue) from WT 12 months old, rd9 12 months old and rd9 18 months old mice. Scale bar is 20 μm. Highly reactive Muller cells’ processes are visible at in rd912 months and rd9 18 months. INL, Inner Nuclear Layer; GCL, Ganglion cell layer.

We found that Muller cells processes exhibit only a moderate GFAP upregulation (with radial processes more intensely immunoreactive) in rd9 12 months compared to WT controls (Figure 9). Such reactivity becomes slightly more diffused across the retina in rd9 18 months compared to rd9 12 months (Figure 9) but never extends to astrocytes in the time span considered in this study (Figure 9).

**FIGURE 10:**
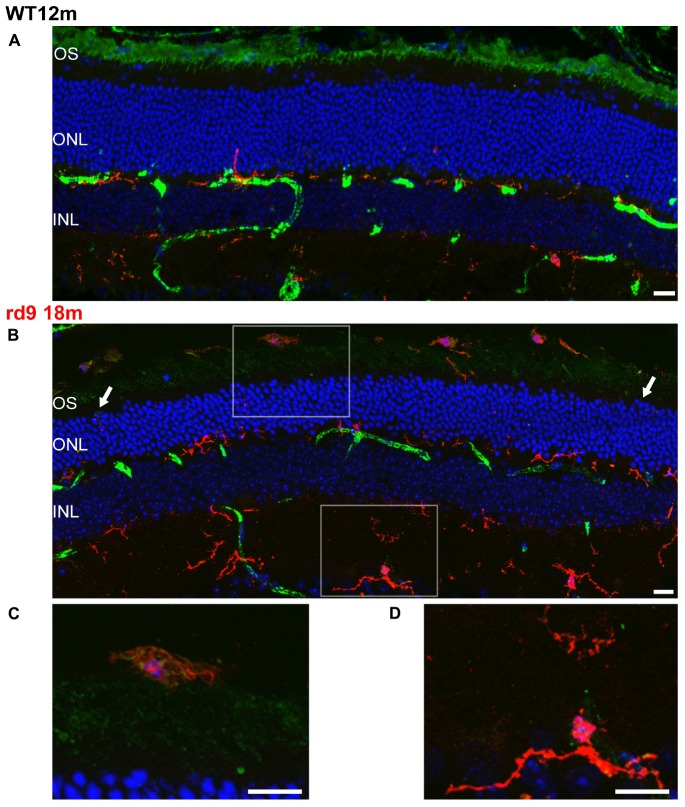
Rod Outer Segments and Microglial reactivity in the outer retina. **(A,B)** Representative image of vertical retinal sections stained with anti-Light Sensitive Channel (green), anti-Iba1 (red) antibodies and Dapi (blue). A: WT 12 months old mouse; B: rd9 18 months old mouse. Arrows point to the left and right sides of the section, where the numbers of ONL rows of nuclei are visibly different, indicating local variability in rod degeneration. Scale bar is 20 μm. **(C)** Detail from **(B)**. Note ameboid morphology of microglia in the subretinal space and the presence of LSC positive residues in intracellular inclusions. Scale bar is 10 μm. **(D)** Detail from **(B)**. Note ramified morphology of microglia in the inner retina. Scale bar is 10 μm. OS, Outer Segments; ONL; Outer Nuclear Layer; OPL; Outer Plexiform Layer.

Putative microglia/macrophages were studied through anti-Iba1 antibodies; double immunostaining for the light-sensitive channel (LSC) of rods allowed visualization of the spatial relationship between microglia and outer segments of degenerating photoreceptors (Figure 10). In normal conditions, the ONL is completely void of microglia/macrophages, being resident microglia normally distributed in the two plexiform layers (Figure 10). Rd9 mice showed a remarkable increase in the number of microglial cells/macrophages located at the outer retinal level, in close proximity to rod outer segments (Figure 10 and Supplementary Figures S3A,B). These Iba-1 positive cells had ameboid morphologies (Figures 10B,C), indicative of activated proinflammatory state (Langmann, 2007; Li et al., 2015), and were also observed to contain LSC-positive vacuoles, strongly suggesting their active phagocytosis of dying rod outer segments (Figure 10 and Supplementary Figure S3B). The inner plexus of microglia displayed a complex and ramified morphology (Figure 10), typical of the physiological microglial condition, reinforcing the notion that degeneration in the rd9 mouse model is mostly a process confined to the outer retina.

Interestingly, the presence of microglia/macrophages at the outer retinal level suggests recruitment from the blood vessels, indicative of a generalized inflammatory/immune reaction.

### Retinal Pigment Epithelium (RPE) Alterations

The RPE tight junction network is a key component of the outer BRB, while retinal capillary endothelial cells contribute to the inner BRB (Cunha-Vaz et al., 2011; Campbell et al., 2018). Tight junctions are made up of intracellular, transmembrane and extracellular proteins that allow intercellular contacts and relative intracellular signaling: Zonula Occludens 1 (ZO-1) is an intracellular tight junction scaffolding protein, regulating RPE proliferation, patterning and homeostasis in physiological and pathological conditions (Itoh et al., 1999; González-Mariscal et al., 2003; Georgiadis et al., 2010; Campbell et al., 2018).

**FIGURE 11:**
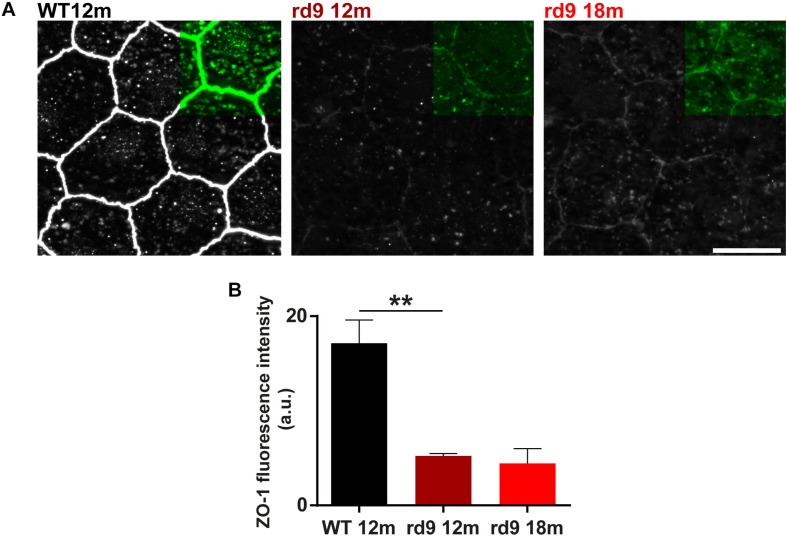
RPE remodeling. **(A)** Representative images of RPE whole mount ICCH with anti-Zonula Occludens 1 (ZO-1) antibodies from WT 12 months old, rd9 12 months old and rd9 18 months old mice. Scale bar is 10 μm. **(B)** Quantification (mean ± se) of Fluorescence intensity (a.u.) in WT 12 months old, rd9 12 months old and rd9 18 months old mice. ^∗∗^for *p* < 0.005.

We investigated RPE morphology through ZO-1 immunostaining (Figure 11). Qualitative exploratory analysis disclosed a critical decrement of the intensity of ZO-1 staining in rd9 mice with respect to WT controls (Figure 11A). Quantification confirmed the almost 4-fold, decrease in ZO-1 immunofluorescence in rd9 12 months [5.2 ± 0.3 arbitrary units (a.u.)] compared to their WT counterpart (17.14 ± 2.4 a.u.) (*p* = 0.003), with changes being maintained also in rd9 18 months (4.4 ± 1.5 a.u.) (Figure 11B).

These findings suggest that RPE integrity is critically altered in rd9 mutants, pinpointing at a non-linearity in the RPE changes with respect to photoreceptor loss.

## Discussion

In this study, we provide the secondary characterization of the retinal phenotype of the rd9 mouse model of XLRP, originally described by Thompson et al. (2012), carrying a mutation in the RPGR-ORF15 gene.

Even though both rods and cones carry the mutation, degeneration in this mouse model is mainly driven by rod death, with survival of 65% of all photoreceptors and 100% of cones at 1 year of age compared to WT mice. Degeneration occurs in a patchy fashion with a measurable intraretinal variation. This feature could not be clearly ascribed to a topographical pattern of photoreceptor loss, nonetheless, together with cone preservation, it is a recurrent finding in XLRP patients (Charng et al., 2016).

The slow but continuous process of rod loss entails an expected remodeling in post synaptic neurons, with thickening and extension toward the outer retina in horizontal cells axonal arborizations and major dendritic sprouting of rod bipolar cells. Sprouting is paired by dislocation of rod spherules (but not cone pedicles) in the outer retina, where they are found at different depths throughout the ONL. Hence, it appears that synaptic connections of rods and their partners in the OPL are maintained, albeit spatially rearranged. These changes might be due to excessive and dysregulated release of neurotransmitter during rod death, working as a growth signal for second-order neurons (McKinney et al., 2002). Dendritic retraction of second order neurons, typically observed in faster forms of retinal degeneration (Jones et al., 2003; Marc et al., 2003; Gargini et al., 2007; Strettoi, 2015), is never observed in rd9 mutants, reinforcing the notion that only major photoreceptor death can drive synaptic deafferentation in the outer retina and major regressive remodeling of inner retinal neurons and suggesting the existence of a photoreceptor loss threshold for initiating this remodeling.

Cone pedicles maintain their spatial location but undergo slight enlargement, paralleled by remodeling of horizontal cells dendrites. In the face of these changes, cone bipolar cells are spared, as assessed by morphological examination of types 2, 3, 4, 5, 6 cone bipolar cells. The complete survival of cones and their minor changes at the synaptic level despite the mutation suggests that these cells are less metabolically vulnerable than rods, perhaps as a consequence of the slower or absent renewal of outer segments (Young, 1971; Hogan et al., 1974; Anderson et al., 1980). Secondly, remaining rods may maintain a relatively normal outer retinal microenvironment halting the death of even vulnerable cones (Aït-Ali et al., 2015). Furthermore, cone conservation might partly be responsible for the overall preservation of retinal architecture (Jones et al., 2016). In agreement with morphological data, the scotopic ERG records a reduction of both the a-wave measured 7 ms post stimulus and of the b-wave, confirming the loss of rod photoreceptors and a malfunctioning of the synapses with rod bipolar cells. Despite the considerable maintenance of cone morphological integrity and number, ERG data demonstrate abnormalities in the physiology of the cone system, indicated by the reduction of photopic b-wave as well as by the decrement in OPs. The latter are indicative of a worsening of the transfer of information from photoreceptors to inner retinal neurons. ERG data, also showing delayed kinetics of both scotopic and photopic responses, confirm and extend previous studies on the same animal model (Thompson et al., 2012) and correlate with existing literature on other paradigms of retinal degeneration (Gargini et al., 2007; Piano et al., 2016; Tanabu et al., 2019) all demonstrating that alterations of retinal physiology can be detected before any major morphological change besides rod loss.

The overall process of glial activation and GFAP upregulation typical of RP (Marc et al., 2003; Gargini et al., 2007; Jones et al., 2016) appears limited in the rd9 phenotype, with mild activation of Muller cell processes and presence of infiltrating and/or migrating microglia/macrophages in the outer retina and mostly at the subretinal space. Compared to mutants where the degeneration takes place in few weeks, this phenotype is sensibly milder (Gargini et al., 2007; Zhao et al., 2015; Guadagni et al., 2019). Despite relatively moderate changes in other retinal cell types, RPE cells visibly down-regulate ZO-1, a scaffolding protein, physiologically responsible of epithelium proliferation control and BRB permeability (Itoh et al., 1999; González-Mariscal et al., 2003; Georgiadis et al., 2010). Changes in the RPE junctional architecture have been previously characterized in other models of retinal degeneration (Campbell et al., 2006; Chrenek et al., 2012), nonetheless, to our knowledge, this is the first report of a decrease of ZO-1 immunostaining in an RP model, suggesting an increase of BRB permeability. Indeed, increases in BRB permeability are common in RP patients (Mallick et al., 1984; van Dorp et al., 1992) and also in diabetic retinopathy and Age-related Macular Degeneration (AMD) (Cunha-Vaz et al., 2011; Cunha-Vaz, 2017). Their role in RP progression remains unknown and worth investigating. This suggests that the rd9 mutant can be used to reveal abnormalities of the outer BRB and to study their possible pathological role, in the presence of relatively conserved retinal architecture, mirroring early stages of disease or slowly progressing phenotypes. Generalization of these findings to other mouse models of RP would support the search of mutation-independent biomarkers of early stage disease and motivate development of new therapeutic strategies to halt BRB changes.

Altogether, observed changes occurring in rd9 retinas recapitulate very early stages of degeneration in other mouse models of RP and in RP patients (Strettoi and Pignatelli, 2000; Strettoi et al., 2002, 2003; Marc et al., 2003; Pignatelli et al., 2004; Gargini et al., 2007; Strettoi, 2015; Jones et al., 2016). Indeed, increased or misplaced wiring of inner retinal neurons has been observed in human retinal degeneration and in mouse models at very early stages of the disease. In most cases, it is quickly followed by dendritic retraction and regressive remodeling of second orderd retinal neurons (Table 2; Milam et al., 1998; Fariss et al., 2000; Strettoi et al., 2002; Jones et al., 2003, 2016; Marc et al., 2003; Pignatelli et al., 2004). This regressive remodeling is predicted to take place only around 30 months of age in rd9 mice, when ONL thickness is relevantly reduced (Chang et al., 2002). This highlights the potential of the rd9 mouse model as a tool to study early phases of retinal degeneration in a slow-progressing phenotype, with the aim of searching for disease biomarkers (e.g., BRB alterations or opsin mislocalization (Chang et al., 2002; Thompson et al., 2012). This could accelerate diagnosis in human patients and develop treatments to be used in contexts where retinal architecture is largely conserved, like targeted gene therapy, indeed a planned treatment for X-linked RP (Boye et al., 2013; Aguirre, 2017; DiCarlo et al., 2018; Trapani and Auricchio, 2018).

**TABLE 2:** Remodeling of retinal cell types at early stages of degeneration in different mouse models.

	**Horizontal cells**	**Rod bipolars**	**Cone bipolars**	**Muller cells**	**Microglia**
rd9 mouse (12/18 months)	Dendritic and axonal sprouting toward the ONL	Dendritic sprouting toward the ONL	Preserved morphology and synaptic architecture	Slightly abnormal GFAP expression and reactivity	Migration in the outer retina and transition to activated ameboid morphology in the outer retina
XLPRA2-affected dogs (< 42 weeks)	Decreased complexity of thin synaptic endings and following dendritic retraction	Dendritic retraction	Preserved morphology and synaptic architecture	Abnormal GFAP expression and reactivity	N/A
rd10 mouse (< P45)	Decreased complexity of thin synaptic endings and following dendritic retraction	Dendritic retraction and mislocalization of metabotropic glutamate receptors	Preserved morphology and synaptic architecture followed by dendritic retraction	Abnormal GFAP expression and reactivity	Migration in the outer retina and transition to activated ameboid morphology
Crx^–/–^ mouse (5–8 months)	Axonal sprouting toward the INL	Dendritic retraction and mislocalization of metabotropic glutamate receptors	Preserved morphology and synaptic architecture followed by dendritic retraction	N/A	N/A
Tvrm4 (7 days post-induction)	Dendritic retraction and axonal arbor thickening	Dendritic retraction and mislocalization of metabotropic glutamate receptors	Dendritic retraction	Abnormal GFAP expression and reactivity	Migration in the outer retina and transition to activated ameboid morphology
P23H transgenic rat (< P40)	Decreased complexity of thin synaptic endings and following dendritic retraction	Dendritic retraction and mislocalization of metabotropic glutamate receptors	Preserved morphology and synaptic architecture followed by dendritic retraction	Abnormal GFAP expression and reactivity	Migration in the outer retina and transition to activated ameboid morphology

*References are: XLPRA2 affected dogs (Beltran et al., 2006); rd10 mouse (Gargini et al., 2007; Zabel et al., 2016); Crx^–/–^ (Pignatelli et al., 2004); Tvrm4 mouse (Gargini et al., 2017); P23H rat (Cuenca et al., 2004; Fernández-Sánchez et al., 2015; Noailles et al., 2016). N/A, not available.*

## Data Availability

Protocols and analytic methods are given in the text. The datasets generated are available upon request to the corresponding author.

## Ethics Statement

The animal study was reviewed and approved by the CNR Neuroscience Institute, Pisa, Animal Welfare Ethical Committee; and Italian Ministry of Health Protocol #17/E-2017, Authorization 599 2017-PR (for CNR Neuroscience Institute, Pisa) and by the Ethical Committee for Animal Welfare, University of Pisa; and Italian Ministry of Health, Protocol DGSAF0001996/2014, Authorization 653/2017-PR (Department of Pharmacy, University of Pisa).

## Author Contributions

All authors conceived the study. AF, MB, and EN performed the histological experiments and collected the data. MB conducted the first original observations on RPE abnormalities. AF and ES performed the data analysis. CG and IP performed the electroretinogram recordings, and collected and analyzed the data. AF, CG, and ES wrote the manuscript.

## Conflict of Interest Statement

The authors declare that the research was conducted in the absence of any commercial or financial relationships that could be construed as a potential conflict of interest.

## Footnotes

1 https://sph.uth.edu/retnet/

## References

[B1] Aguirre G. D. (2017). Concepts and strategies in retinal gene therapy. *Investig. Ophthalmol. Vis. Sci.* 58 5399–5411. 10.1167/iovs.17-22978 29053763 PMC5656413

[B2] Aït-Ali N. Fridlich R. Millet-Puel G. Clérin E. Delalande F. Jaillard C. (2015). Rod-derived cone viability factor promotes cone survival by stimulating aerobic glycolysis. *Cell* 161 817–832. 10.1016/j.cell.2015.03.023 25957687

[B3] Anderson D. H. Fisher S. K. Erickson P. A. Tabor G. A. (1980). Rod and cone disc shedding in the rhesus monkey retina: a quantitative study. *Exp. Eye Res.* 30 559–574. 10.1016/0014-4835(80)90040-8 7409012

[B4] Barone I. Novelli E. Piano I. Gargini C. Strettoi E. (2012). environmental enrichment extends photoreceptor survival and visual function in a mouse model of Retinitis Pigmentosa. *PLoS One* 7:e50726. 10.1371/journal.pone.0050726 23209820 PMC3508993

[B5] Beltran W. A. Hammond P. Acland G. M. Aguirre G. D. (2006). A Frameshift Mutation in RPGR Exon ORF15 causes photoreceptor degeneration and inner retina remodeling in a model of X-Linked Retinitis Pigmentosa. *Invest. Ophthalmol. Vis. Sci.* 47 1669–1681. 10.1167/iovs.05-0845 16565408

[B6] Berntson A. K. Morgans C. W. (2005). Distribution of the presynaptic calcium sensors, synaptotagmin I/II and synaptotagmin III, in the goldfish and rodent retinas. *J. Vis.* 3 274–280. 10.1167/3.4.3 12803536

[B7] Besharse J. C. Hollyfield J. G. Rayborn M. E. (1977). Turnover of rod photoreceptor outer segments: II. membrane addition and loss in relationship to light. *J. Cell Biol.* 75 507–527. 10.1083/jcb.75.2.507 264121 PMC2109927

[B8] Boye S. E. Boye S. L. Lewin A. S. Hauswirth W. W. (2013). A comprehensive review of retinal gene therapy. *Mol. Ther.* 21 509–519. 10.1038/mt.2012.280 23358189 PMC3642288

[B9] Burgoyne T. Meschede I. P. Burden J. J. Bailly M. Seabra M. C. Futter C. E. (2015). Rod disc renewal occurs by evagination of the ciliary plasma membrane that makes cadherin-based contacts with the inner segment. *Proc. Natl. Acad. Sci. U.S.A.* 112 15922–15927. 10.1073/pnas.1509285113 26668363 PMC4702997

[B10] Campbell M. Cassidy P. S. O’Callaghan J. Crosbie D. E. Humphries P. (2018). Manipulating ocular endothelial tight junctions: applications in treatment of retinal disease pathology and ocular hypertension. *Prog. Retin. Eye Res.* 62 120–133. 10.1016/j.preteyeres.2017.09.003 28951125

[B11] Campbell M. Humphries M. Kennan A. Kenna P. Humphries P. Brankin B. (2006). Aberrant retinal tight junction and adherens junction protein expression in an animal model of autosomal dominant Retinitis pigmentosa: the Rho(−/−) mouse. *Exp. Eye Res.* 83 484–492. 10.1016/j.exer.2006.01.032 16643895

[B12] Chadha A. Volland S. Baliaouri N. V. Tran E. M. Williams D. S. (2019). The route of the visual receptor rhodopsin along the cilium. *J. Cell Sci.* 132:jcs229526. 10.1242/jcs.229526 30975916 PMC6550008

[B13] Chang B. Hawes N. L. Hurd R. E. Davisson M. T. Nusinowitz S. Heckenlively J. R. (2002). Retinal degeneration mutants in the mouse. *Vis. Res.* 42 517–525. 10.1016/S0042-6989(01)00146-8 11853768

[B14] Charng J. Cideciyan A. V. Jacobson S. G. Sumaroka A. Schwartz S. B. Swider M. (2016). Variegated yet non-random rod and cone photoreceptor disease patterns in RPGR-ORF15-associated retinal degeneration. *Hum. Mol. Genet.* 25 5444–5459. 10.1093/hmg/ddw361 27798110 PMC6078602

[B15] Chrenek M. A. Dalal N. Gardner C. Grossniklaus H. Jiang Y. Boatright J. H. (2012). Analysis of the RPE sheet in the rd10 retinal degeneration model. *Adv. Exp. Med. Biol.* 723 641–647. 10.1007/978-1-4614-0631-0_81 22183388 PMC3732179

[B16] Cuenca N. Pinilla I. Sauvé Y. Lu B. Wang S. Lund R. D. (2004). Regressive and reactive changes in the connectivity patterns of rod and cone pathways of P23H transgenic rat retina. *Neuroscience* 127 301–317. 10.1016/j.neuroscience.2004.04.042 15262321

[B17] Cunha-Vaz J. (2017). The blood-retinal barrier in the management of retinal disease: EURETINA award lecture. *Ophthalmologica* 237 1–10. 10.1159/000455809 28152535

[B18] Cunha-Vaz J. Bernardes R. Lobo C. (2011). Blood-retinal barrier. *Eur. J. Ophthalmol.* 21 3–9. 10.5301/EJO.2010.6049 23264323

[B19] Daiger S. P. Bowne S. J. Sullivan L. S. (2007). Perspective on genes and mutations causing retinitis pigmentosa. *Arch. Ophthalmol.* 125 151–158. 10.1001/archopht.125.2.151 17296890 PMC2580741

[B20] Deczkowska A. Amit I. Schwartz M. (2018). Microglial immune checkpoint mechanisms. *Nat. Neurosci.* 21 779–786. 10.1038/s41593-018-0145-x 29735982

[B21] DiCarlo J. E. Mahajan V. B. Tsang S. H. (2018). Gene therapy and genome surgery in the retina. *J. Clin. Invest.* 128 2177–2188. 10.1172/JCI120429 29856367 PMC5983345

[B22] Fariss R. N. Li Z. Y. Milam A. H. (2000). Abnormalities in rod photoreceptors, amacrine cells, and horizontal cells in human retinas with retinitis pigmentosa. *Am. J. Ophthalmol.* 129 215–223. 10.1016/S0002-9394(99)00401-8 10682975

[B23] Fernández-Sánchez L. Lax P. Campello L. Pinilla I. Cuenca N. (2015). Astrocytes and müller cell alterations during retinal degeneration in a transgenic rat model of Retinitis Pigmentosa. *Front. Cell. Neurosci.* 9:484. 10.3389/fncel.2015.00484 26733810 PMC4686678

[B24] Fletcher E. Phipps J. Ward M. Puthussery T. Wilkinson-Berka J. (2007). Neuronal and glial cell abnormality as predictors of progression of diabetic retinopathy. *Curr. Pharm. Des.* 13 2699–2712. 10.2174/138161207781662920 17897014

[B25] Gargini C. Novelli E. Piano I. Biagioni M. Strettoi E. (2017). Pattern of retinal morphological and functional decay in a light-inducible, rhodopsin mutant mouse. *Sci. Rep.* 7:5730. 10.1038/s41598-017-06045-x 28720880 PMC5516022

[B26] Gargini C. Terzibasi E. Mazzoni F. Strettoi E. (2007). Retinal organization in the retinal degeneration 10 (rd10) mutant mouse: a morphological and ERG study. *J. Comp. Neurol.* 500 222–238. 10.1002/cne.21144 17111372 PMC2590657

[B27] Georgiadis A. Tschernutter M. Bainbridge J. W. B. Balaggan K. S. Mowat F. West E. L. (2010). The tight junction associated signalling proteins ZO-1 and ZONAB regulate retinal pigment epithelium homeostasis in mice. *PLoS One* 5:e15730. 10.1371/journal.pone.0015730 21209887 PMC3012699

[B28] González-Mariscal L. Betanzos A. Nava P. Jaramillo B. E. (2003). Tight junction proteins. *Prog. Biophys. Mol. Biol.* 81 1–44. 10.1016/S0079-6107(02)00037-8 12475568

[B29] Gonzälez-soriano J. (1994). Morphological types of horizontal cell in rodent retinae: a comparison of rat, mouse, gerbil, and guinea pig. *Vis. Neurosci.* 11 501–517. 10.1017/S095252380000242X 8038125

[B30] Guadagni V. Biagioni M. Novelli E. Aretini P. Mazzanti C. M. Strettoi E. (2019). Rescuing cones and daylight vision in retinitis pigmentosa mice. *FASEB J.* 33 10177–10192. 10.1096/fj.201900414R 31199887 PMC6764477

[B31] Hancock H. A. Kraft T. W. (2004). Oscillatory potential analysis and ERGs of normal and diabetic rats. *Investig. Ophthalmol. Vis. Sci.* 45 1002–1008. 10.1167/iovs.03-1080 14985323

[B32] Hartong D. T. Berson E. L. Dryja T. P. (2006). Retinitis pigmentosa. *Lancet* 368 1795–1809. 10.1016/S0140-6736(06)69740-7 17113430

[B33] Hogan M. J. Wood I. Steinberg R. H. (1974). Phagocytosis by pigment epithelium of human retinal cones. *Nature* 252 305–307. 10.1038/252305a0 4431450

[B34] Huang W. C. Wright A. F. Roman A. J. Cideciyan A. V. Manson F. D. Gewaily D. Y. (2012). RPGR-associated retinal degeneration in human X-linked RP and a murine model. *Investig. Ophthalmol. Vis. Sci.* 53 5594–5608. 10.1167/iovs.12-10070 22807293 PMC3422104

[B35] Itoh M. Furuse M. Morita K. Kubota K. Saitou M. Tsukita S. (1999). Direct binding of three tight junction-associated MAGUKs, ZO-1, ZO-2, and ZO-3, with the COOH termini of claudins. *J. Cell Biol.* 147 1351–1363. 10.1083/jcb.147.6.1351 10601346 PMC2168087

[B36] Jones B. W. Pfeiffer R. L. Ferrell W. D. Watt C. B. Marmor M. Marc R. E. (2016). Retinal remodeling in human retinitis pigmentosa. *Exp. Eye Res.* 150 149–165. 10.1016/j.exer.2016.03.018 27020758 PMC5031517

[B37] Jones B. W. Watt C. B. Frederick J. M. Baehr W. Chen C. K. Levine E. M. (2003). Retinal remodeling triggered by photoreceptor degenerations. *J. Comp. Neurol.* 464 1–16. 10.1002/cne.10703 12866125

[B38] Kim D. S. Ross S. E. Trimarchi J. M. Aach J. Greenberg M. E. Cepko C. L. (2008). Identification of molecular markers of bipolar cells in the murine retina. *J. Comp. Neurol.* 507 1795–1810. 10.1002/cne.21639 18260140 PMC2665264

[B39] Langmann T. (2007). Microglia activation in retinal degeneration. *J. Leukoc. Biol.* 81 1345–1351. 10.1189/jlb.0207114 17405851

[B40] Lei B. Yao G. Zhang K. Hofeldt K. J. Chang B. (2006). Study of rod- and cone-driven oscillatory potentials in mice. *Investig. Ophthalmol. Vis. Sci.* 47 2732–2738. 10.1167/iovs.05-1461 16723493

[B41] Li L. Eter N. Heiduschka P. (2015). The microglia in healthy and diseased retina. *Exp. Eye Res.* 136 116–130. 10.1016/j.exer.2015.04.020 25952657

[B42] Liddelow S. A. Guttenplan K. A. Clarke L. E. Bennett F. C. Bohlen C. J. Schirmer L. (2017). Neurotoxic reactive astrocytes are induced by activated microglia. *Nature* 541 481–487. 10.1038/nature21029 28099414 PMC5404890

[B43] Lyraki R. Megaw R. Hurd T. (2016). Disease mechanisms of X-linked retinitis pigmentosa due to RP2 and RPGR mutations. *Biochem. Soc. Trans.* 44 1235–1244. 10.1042/bst20160148 27911705

[B44] Mallick K. S. Zeimer R. C. Fishman G. A. Blair N. P. Anderson R. J. (1984). Transport of fluorescein in the ocular posterior segment in Retinitis Pigmentosa. *Arch. Ophthalmol.* 102 691–696. 10.1001/archopht.1984.01040030547013 6721754

[B45] Marc R. E. Jones B. W. Watt C. B. Strettoi E. (2003). Neural remodeling in retinal degeneration. *Prog. Retin. Eye Res.* 22 607–655. 10.1016/S1350-9462(03)00039-9 12892644

[B46] Marmorstein A. D. Marmorstein L. Y. Sakaguchi H. Hollyfield J. G. (2002). Spectral profiling of autofluorescence associated with lipofuscin, Bruch’s membrane, and sub-RPE deposits in normal and AMD eyes. *Investig. Ophthalmol. Vis. Sci.* 43 2435–2441. 12091448

[B47] McKinney R. A. Luthi A. Bandtlow C. E. Gahwiler B. H. Thompson S. M. (2002). Selective glutamate receptor antagonists can induce or prevent axonal sprouting in rat hippocampal slice cultures. *Proc. Natl. Acad. Sci. U.S.A.* 96 11631–11636. 10.1073/pnas.96.20.11631 10500228 PMC18085

[B48] Megaw R. D. Soares D. C. Wright A. F. (2015). RPGR: its role in photoreceptor physiology, human disease, and future therapies. *Exp. Eye Res.* 138 32–41. 10.1016/j.exer.2015.06.007 26093275 PMC4553903

[B49] Milam A. H. Li Z. Y. Fariss R. N. (1998). Histopathology of the human retina in retinitis pigmentosa. *Prog. Retin. Eye Res.* 17 175–205. 10.1016/S1350-9462(97)00012-8 9695792

[B50] Noailles A. Maneu V. Campello L. Gómez-Vicente V. Lax P. Cuenca N. (2016). Persistent inflammatory state after photoreceptor loss in an animal model of retinal degeneration. *Sci. Rep.* 6:33356. 10.1038/srep33356 27624537 PMC5022039

[B51] Piano I. Novelli E. Della Santina L. Strettoi E. Cervetto L. Gargini C. (2016). Involvement of autophagic pathway in the progression of retinal degeneration in a mouse model of diabetes. *Front. Cell. Neurosci.* 10:42. 10.3389/fncel.2016.00042 26924963 PMC4759287

[B52] Pignatelli V. Cepko C. L. Strettoi E. (2004). Inner retinal abnormalities in a mouse model of Leber’s Congenital Amaurosis. *J. Comp. Neurol.* 469 351–359. 10.1002/cne.11019 14730587

[B53] Puthussery T. Gayet-Primo J. Taylor W. R. (2010). Localization of the calcium-binding protein secretagogin in cone bipolar cells of the mammalian retina. *J. Comp. Neurol.* 518 513–525. 10.1002/cne.22234 20020539 PMC3855033

[B54] Rao K. N. Anand M. Khanna H. (2016). The carboxyl terminal mutational hotspot of the ciliary disease protein RPGR ORF15 (retinitis pigmentosa GTPase regulator) is glutamylated in vivo. *Biol. Open* 5 424–428. 10.1242/bio.016816 26941104 PMC4890669

[B55] Silverman S. M. Wong W. T. (2018). Microglia in the retina: roles in development, maturity, and disease. *Annu. Rev. Vis. Sci.* 4 45–77. 10.1146/annurev-vision-091517-034425 29852094

[B56] Strettoi E. (2015). A survey of retinal remodeling. *Front. Cell. Neurosci.* 9:494. 10.3389/fncel.2015.00494 26778960 PMC4688370

[B57] Strettoi E. Pignatelli V. (2000). Modifications of retinal neurons in a mouse model of retinitis pigmentosa. *Proc. Natl. Acad. Sci. U.S.A.* 97 11020–11025. 10.1073/pnas.190291097 10995468 PMC27141

[B58] Strettoi E. Pignatelli V. Rossi C. Porciatti V. Falsini B. (2003). Remodeling of second-order neurons in the retina of rd/rd mutant mice. *Vis. Res.* 43 867–877. 10.1016/S0042-6989(02)00594-1 12668056

[B59] Strettoi E. Porciatti V. Falsini B. Pignatelli V. Rossi C. (2002). Morphological and functional abnormalities in the inner retina of the rd/rd mouse. *J. Neurosci.* 22 5492–5504. 10.1523/jneurosci.22-13-05492.2002 12097501 PMC6758223

[B60] Tanabu R. Sato K. Monai N. Yamauchi K. Gonome T. Xie Y. (2019). The findings of optical coherence tomography of retinal degeneration in relation to the morphological and electroretinographic features in RPE65 -/- mice. *PLoS One* 14:e0210439. 10.1371/journal.pone.0210439 30695025 PMC6350961

[B61] Thompson D. A. Khan N. W. Othman M. I. Chang B. Jia L. Grahek G. (2012). Rd9 is a naturally occurring mouse model of a common form of retinitis pigmentosa caused by mutations in RPGR-ORF15. *PLoS One* 7:e35865. 10.1371/journal.pone.0035865 22563472 PMC3341386

[B62] Trapani I. Auricchio A. (2018). Seeing the light after 25 Years of retinal gene therapy. *Trends Mol. Med.* 24 669–681. 10.1016/j.molmed.2018.06.006 29983335

[B63] Tsang S. H. Sharma T. (2018). X-linked retinitis pigmentosa. *Adv. Exp. Med. Biol.* 1085 31–35. 10.1007/978-3-319-95046-4_8 30578481

[B64] van Dorp D. B. Wright A. F. Carothers A. D. Bleeker-Wagemakers E. M. (1992). A family with RP3 type of X-linked retinitis pigmentosa: an association with ciliary abnormalities. *Hum. Genet.* 88 331–334. 10.1007/BF00197269 1733835

[B65] Wassle H. Puller C. Muller F. Haverkamp S. (2009). Cone contacts, mosaics, and territories of bipolar cells in the mouse retina. *J. Neurosci.* 29 106–117. 10.1523/jneurosci.4442-08.2009 19129389 PMC6664901

[B66] Wolfrum U. Schmitt A. (2000). Rhodopsin transport in the membrane of the connecting cilium of mammalian photoreceptor cells. *Cell Motil. Cytoskeleton* 46 95–107. 10.1002/1097-0169(200006)46:2<95::AID-CM2<3.0.CO;2-Q 10891855

[B67] Wright A. F. Chakarova C. F. Abd El-Aziz M. M. Bhattacharya S. S. (2010). Photoreceptor degeneration: genetic and mechanistic dissection of a complex trait. *Nat. Rev. Genet.* 11 273–284. 10.1038/nrg2717 20212494

[B68] Young R. W. (1971). The renewal of rod and cone outer segments in the rhesus monkey. *J. Cell Biol.* 49 303–318. 10.1083/jcb.49.2.303 19866760 PMC2108322

[B69] Zabel M. K. Zhao L. Zhang Y. Gonzalez S. R. Ma W. Wang X. (2016). Microglial phagocytosis and activation underlying photoreceptor degeneration is regulated by CX3CL1-CX3CR1 signaling in a mouse model of retinitis pigmentosa. *Glia* 64 1479–1491. 10.1002/glia.23016 27314452 PMC4958518

[B70] Zhang Q. Giacalone J. C. Searby C. Stone E. M. Tucker B. A. Sheffield V. C. (2019). Disruption of RPGR protein interaction network is the common feature of RPGR missense variations that cause XLRP. *Proc. Natl. Acad. Sci. U.S.A.* 116 1353–1360. 10.1073/pnas.1817639116 30622176 PMC6347721

[B71] Zhao L. Zabel M. K. Wang X. Ma W. Shah P. Fariss R. N. (2015). Microglial phagocytosis of living photoreceptors contributes to inherited retinal degeneration. *EMBO Mol. Med.* 7 1179–1197. 10.15252/emmm.201505298 26139610 PMC4568951

