# Supplementary material for: Retinal Phenotype in the rd9 Mutant Mouse, a Model of X-Linked RP
Source: Front Neurosci. 2019 Sep 19;13:991. doi: 10.3389/fnins.2019.00991 (PMC6761883; doi:10.3389/fnins.2019.00991)
Supplement: Supplementary file 1 [file Table_1.docx]

Supplementary Material

# Spectral profiling of RPE


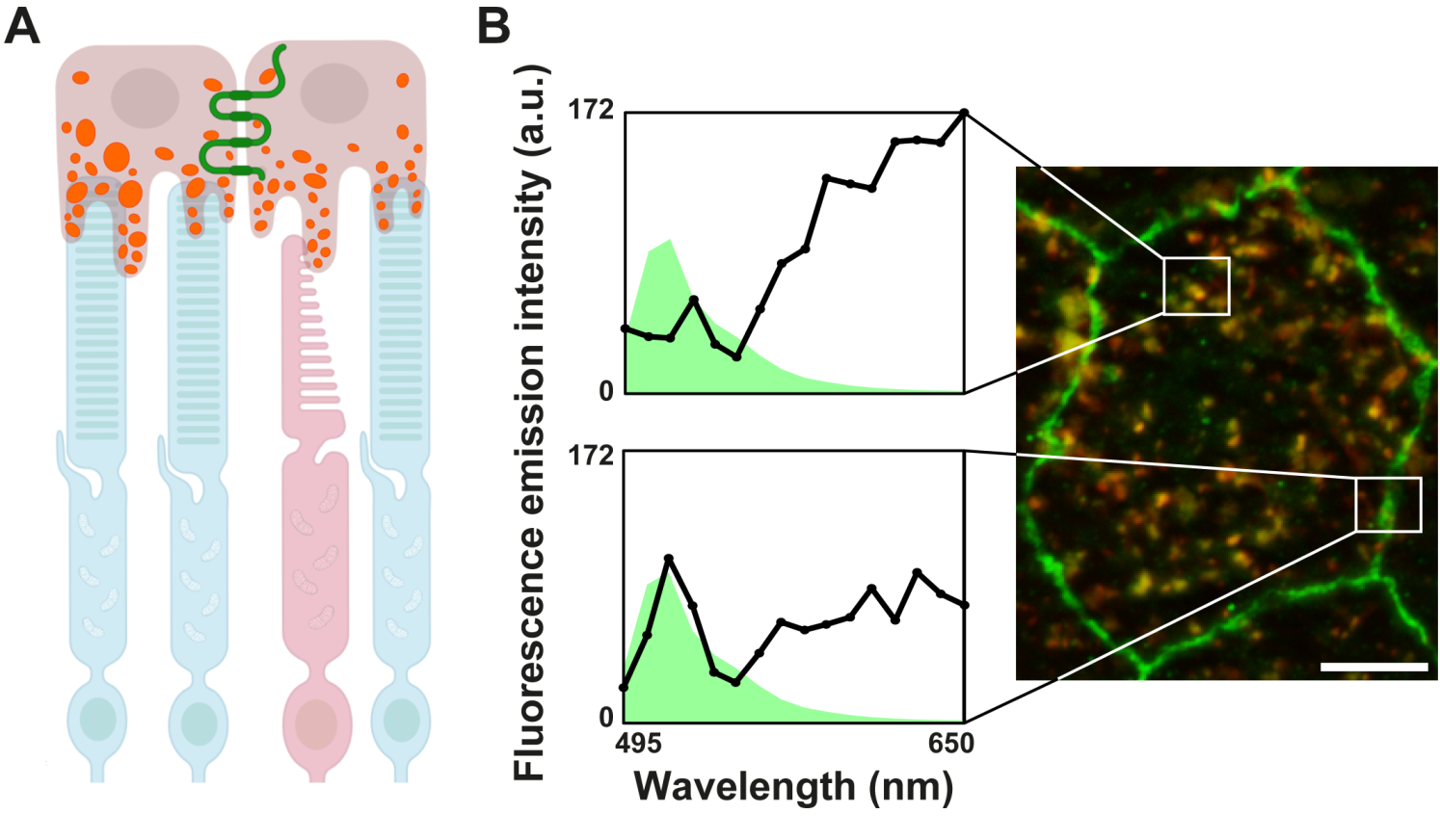


**Supplementary Figure 1.** Spectral profiling of RPE. (A) Schematic drawing of RPE cells and their close relationship with rod and cone outer segments. A tight junction (green) and putative intracellular residues of outer segment metabolism (orange) are represented. (B) Black lines are spectral profiles of autofluorescent residues (upper graph) and Alexa Fluor 488 ZO-1 immunostained profiles (lower graph), both obtained upon 488 nm excitation. Lines have been overlaid on the expected Alexa Fluor 488 spectrum (green) and connected to source components of an RPE cell (right). The latter has been labelled with Zonula Occludens 1 (ZO-1) (green) antibodies and shows autofluorescent (orange) residues. rd9 12 months old mouse. Scale bar is 20μm.

# Automated cone counting algorithm validation

Results obtained from automated counting of single images from retinal whole mount ICCH with anti-cone arrestin antibodies were compared with results obtained from manual counting of the same images trough Pearson correlation analysis. This comparison revealed a significant correlation with an r=0.92 (p<0.0001). (Supp. Fig. 2A).

Normalized error was also measured as:

$$\frac{Manual counting result-Automated Counting result}{Manual counting result}$$

Single image errors were binned in 0.1 intervals and their frequency plotted after fitting with a Gaussian model with mean=0 and standard deviation=0.08 (r=0.99). Error distribution was not significantly skewed thus showing there was no systematic under or overcounting error in algorithm performance.

Together these results suggest our cone counting algorithm is able to quantify retinal cone photoreceptors from z-stacks of their inner segments with an accuracy overlapping that of manual counting procedures.


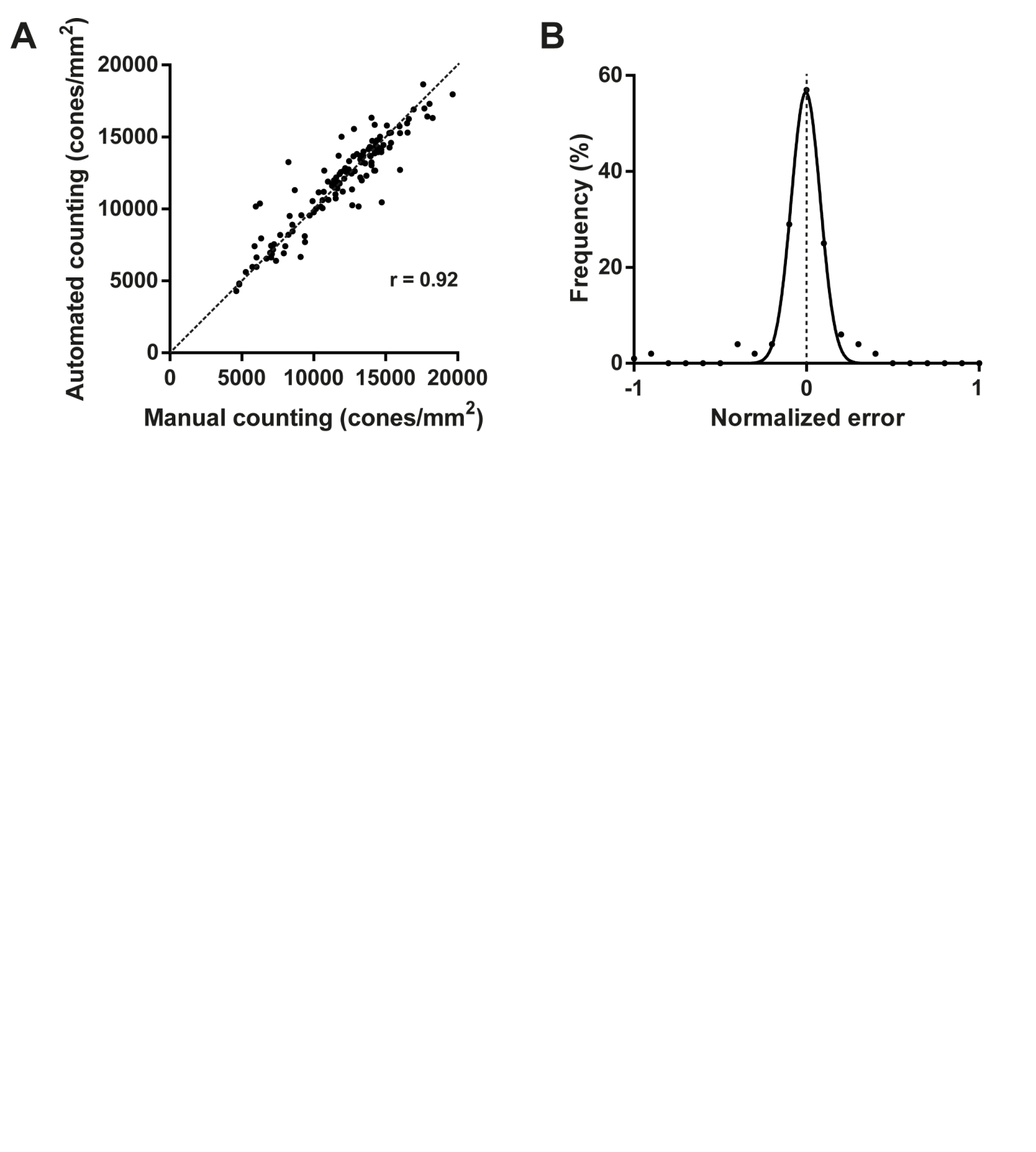


**Supplementary Figure 2.** Validation of the automated cone counting algorithm. (A) Pearson correlation analysis between automated counting and manual counting. For r=1 all dots should lie on the dashed line. (B) Normalized error and fit Gaussian model.

# Microglial activation in rd9 12m

**
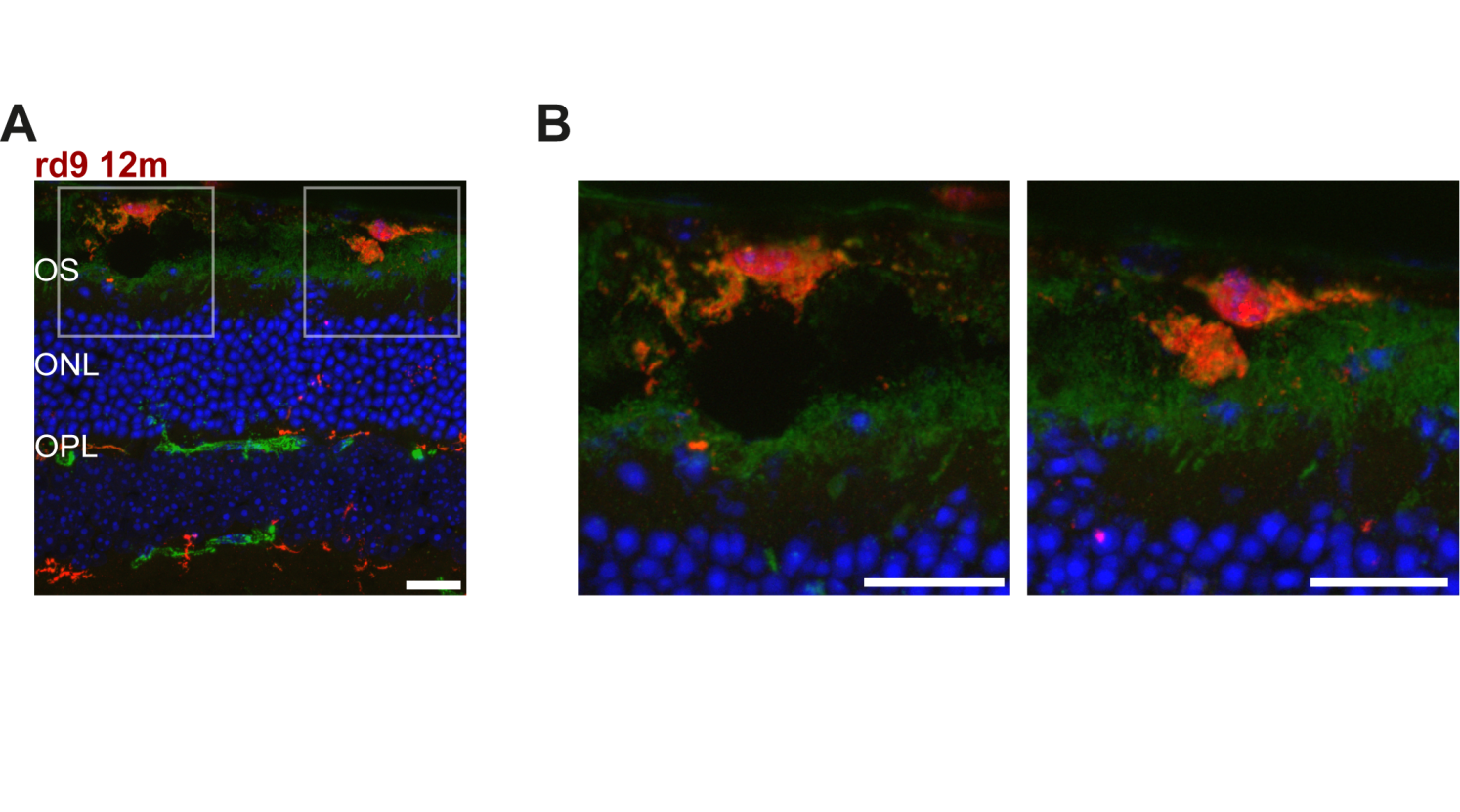
**

**Supplementary Figure 3.** Rod outer segments and microglial reactivity in the outer retina of rd9 12 months old. (A) Representative image of vertical retinal sections stained with anti-light sensitive channel (green), anti-Iba1 (red) antibodies and nuclear staining from WT 12 months old mice. Scale bar is 20 μm. (B) Details from (A). Scale bar is 20 μm. Note ameboid morphology of microglia in the subretinal space and the presence of LSC positive residues in putative intracellular vacuoles. OS: Outer Segments. ONL: Outer Nuclear Layer. OPL: Outer Plexiform Layer.
